# Compact two-port MIMO antenna dual-circularly polarized 28/38 GHz with polarization diversity for 5G and beyond millimeter-wave systems

**DOI:** 10.1038/s41598-026-65078-3

**Published:** 2026-08-11

**Authors:** Asmaa E. Farahat, Khalid. F. A. Hussein

**Affiliations:** Microwave Engineering Department, Electronics Research Institute (ERI), Cairo, Egypt

**Keywords:** Engineering, Physics

## Abstract

This paper presents a compact dual-band circularly polarized two-port MIMO antenna for millimeter-wave applications at 28 and 38 GHz. The single-element antenna occupies an ultra-small area of 8 mm × 16 mm and is fabricated on a Rogers RO3003 substrate (εr = 3, thickness = 0.25 mm). Circular polarization is achieved at both bands, with measured axial ratios of 1.4 dB at 28 GHz and 2.2 dB at 38 GHz, providing robustness against polarization mismatch. The antenna achieves peak realized gains of 7.5 dBi and 5.5 dBi at 28 GHz and 38 GHz, respectively. Experimental results show good impedance matching and radiation patterns in close agreement with simulations. Based on the validated single-element design, a two-port MIMO configuration is implemented, where one element generates RHCP and the other LHCP, enabling polarization diversity. The MIMO antenna exhibits low envelope correlation coefficient and high diversity gain. Owing to its compact size, dual-band circular polarization, and effective polarization-diverse MIMO performance, the proposed antenna is suitable for space-constrained 5G and future millimeter-wave wireless devices.

## Introduction

Millimeter-wave (mm-Wave) frequency bands, particularly 28 GHz and 38 GHz, have emerged as cornerstone candidates for 5G and future wireless communication systems due to their ability to provide large contiguous bandwidths that enable ultra-high data rates, low latency, and enhanced network capacity. These frequency bands have been globally standardized and widely allocated for early 5G deployments, making them highly relevant for both outdoor cellular infrastructure and indoor wireless applications. Nevertheless, operating at mm-Wave frequencies introduces several propagation challenges, including high free-space path loss, atmospheric absorption, and increased susceptibility to blockage and shadowing. As a result, antenna designs for 28/38 GHz systems must simultaneously achieve compact size, sufficient gain, stable radiation characteristics, and high efficiency while remaining compatible with dense integration requirements of modern wireless devices^1–3^.

In this context, circularly polarized (CP) antennas have gained increasing attention for mm-Wave wireless systems. Circular polarization offers significant advantages over linear polarization, including reduced polarization mismatch losses, improved immunity to multipath fading, and relaxed orientation constraints between the transmitter and receiver. These properties are particularly beneficial for mobile terminals, small-cell base stations, and user equipment operating at 28 GHz and 38 GHz, where device orientation and environmental reflections can severely degrade link quality. As a result, CP antennas are considered strong candidates for robust mm-Wave communication links in practical 5G and beyond-5G deployments^4,5^. Sahu et al. proposed a ceramic-based millimeter-wave MIMO antenna for 5G NR Band 261 applications. The antenna operates around 28 GHz and employs a machine-learning-assisted optimization approach to achieve good MIMO performance, including high isolation and low correlation between antenna elements^6^.

Previous research has also focused on circularly polarized MIMO antennas employing advanced decoupling and metasurface techniques to enhance diversity performance^7^. It proposed a 12-port circularly polarized MIMO antenna integrated with a diagonal split-ring metasurface for Sub-6 GHz wireless systems, demonstrating improved isolation and MIMO characteristics through metasurface-assisted design. In another study^8^, introduced a circularly polarized MIMO antenna incorporating parasitic phase control and a cross-loop ground decoupling structure to simultaneously improve polarization purity, isolation, and diversity performance. These studies highlight the growing interest in combining circular polarization with advanced decoupling techniques to achieve high-performance MIMO systems for next-generation wireless communications. Furthermore, a metamaterial Fabry–Perot MIMO antenna with circular polarization was reported in^9^, demonstrating improved gain and diversity performance for 5G and satellite communication systems.

Recent research efforts have focused on realizing compact dual-band CP antennas covering the 28 GHz and 38 GHz bands while maintaining low axial ratio, sufficient gain, and ease of integration. Various techniques have been reported, including modified microstrip patch geometries, dielectric resonator antennas (DRAs), defected ground structures, and metasurface-assisted designs to generate circular polarization at one or both mmWave bands^10–12^. In addition, several works have demonstrated dual-band or multi-band CP antenna arrays operating at 28/38 GHz for beamforming and high-capacity 5G applications, emphasizing the importance of polarization purity and compact size. Despite these advances, achieving dual-band circular polarization within an ultra-compact footprint remains a challenging task, motivating further research on miniaturized CP antenna structures suitable for future mm-Wave wireless devices.

## Circularly-polarized modified quatrefoil single antenna design

A thin, low-loss substrate is chosen to suit the planar configuration of the proposed antenna and its operation in the millimeter-wave band. The antenna is fabricated on a Rogers RO3003™ dielectric material with a thickness of 0.25 mm, relative permittivity of 3, and a loss tangent of 0.001. The antenna geometry and the underlying design methodology are described in detail. Due to the sensitivity of antenna performance to its physical dimensions, the design procedure involves comprehensive parametric optimization to determine the optimal dimensional values. This process ensures efficient operation across the two target frequency bands while maintaining an axial ratio below 3 dB, which is required to achieve high-quality circular polarization in both bands.

To realize circular polarization, the proposed antenna employs a quatrefoil-shaped radiator, whose inherent geometric symmetry supports the generation of circularly polarized fields. In the proposed modified quatrefoil radiator, two orthogonal degenerate modes can be supported within the radiating structure. Circular polarization is obtained by properly perturbing the quatrefoil geometry, such that these two modes are excited with equal amplitudes. The introduced modifications break the structural symmetry in a controlled manner, leading to the excitation of two modes with mutually orthogonal electric-field orientations. One mode exhibits even symmetry, while the other shows odd symmetry with respect to the principal diagonal of the modified quatrefoil. By carefully tuning the geometric parameters of the modified quatrefoil, the required 90° phase difference between the two modes is achieved, resulting in the generation of circular polarization.

Four parasitic elements are placed around the modified quatrefoil-shaped patch and capacitively coupled to it through narrow gaps. This coupling is used to effectively tune the resonant frequencies of the two radiating modes supported by the modified quatrefoil radiator. In addition, narrow slits are introduced in the ground plane to further control the current distribution and enhance impedance matching and polarization performance.m.

**Fig. 1:**
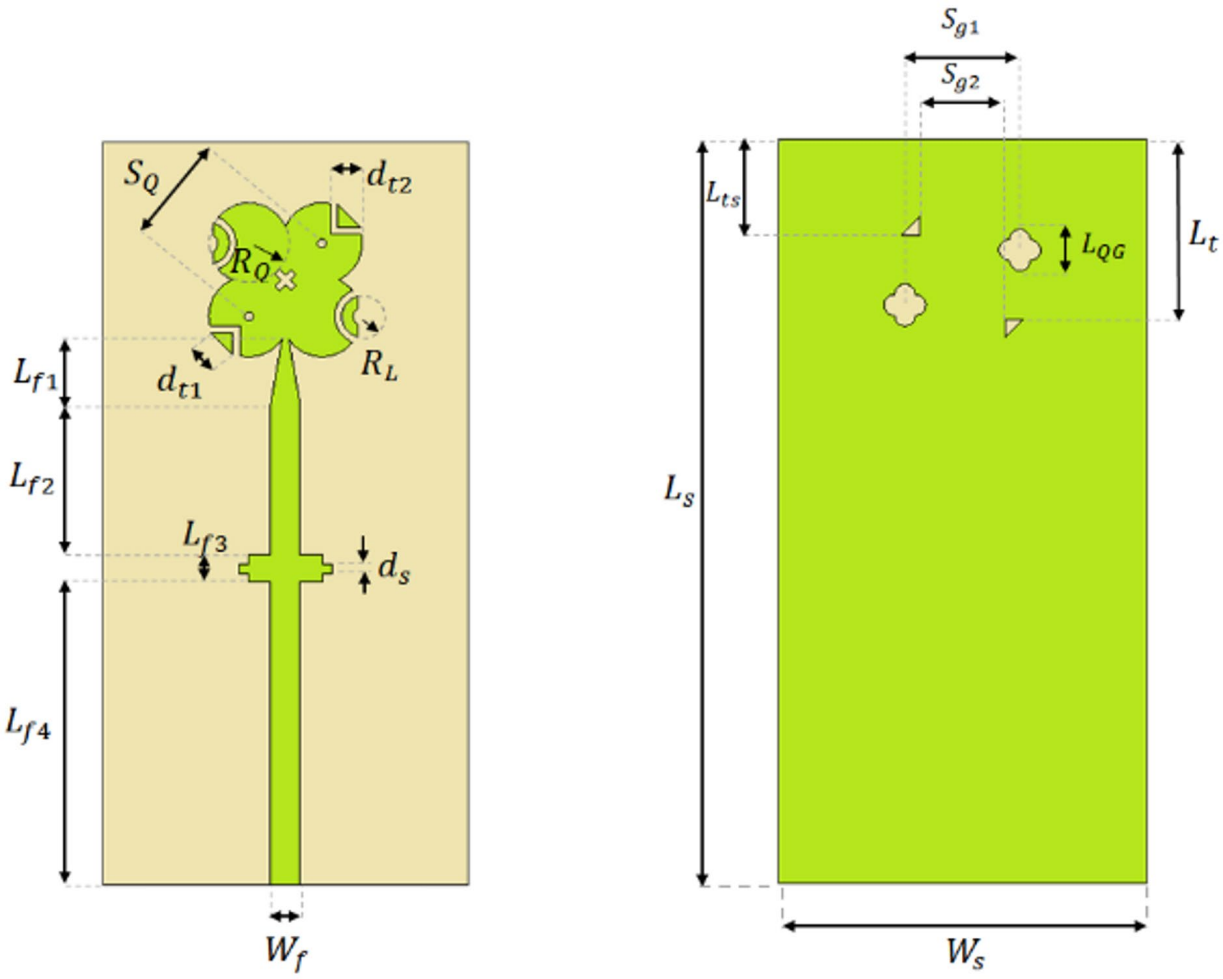
Geometry of the proposed modified quatrefoil-shaped antenna structure.

Impedance matching at both resonant frequencies is realized through careful optimization of the modified quatrefoil patch dimensions, the ground-plane cuts, the four parasitic elements, and the coupling-gap width between the parasitic elements and the main radiator. An inset-fed configuration is intentionally avoided, as it would break the diagonal symmetry of the antenna and negatively impact the circular polarization performance. Instead, the antenna is excited using a tapered microstrip feed line, which preserves structural symmetry while providing effective excitation and input-impedance matching. The tapered microstrip line has a width of 0.64 mm at the feed point. The complete geometry of the proposed antenna, including all relevant structural features, is illustrated in Fig. 1, while the optimized values of the corresponding dimensional parameters used in the design are summarized in Table 1.

**Table 1:** Optimized dimensional parameters of the proposed antenna.

Parameter	$\:{\mathrm{W}}_{\mathrm{f}}$	$\:{\mathrm{L}}_{\mathrm{f}1}$	$\:{\mathrm{L}}_{\mathrm{f}2}$	$\:{\mathrm{L}}_{\mathrm{f}3}$	$\:{\mathrm{L}}_{\mathrm{f}4}$	$\:{\mathrm{d}}_{\mathrm{s}}$	$\:{\mathrm{d}}_{\mathrm{t}1}$	$\:{\mathrm{R}}_{\mathrm{L}}$	$\:{\mathrm{R}}_{\mathrm{Q}}$
Value (mm)	0.64	1.4	3.3	0.6	6.6	0.2	0.71	0.6	0.88
Parameter	$\:{\mathrm{S}}_{\mathrm{Q}}$	$\:{\mathrm{d}}_{\mathrm{t}2}$	$\:{\mathrm{L}}_{\mathrm{s}}$	$\:{\mathrm{W}}_{\mathrm{s}}$	$\:{\mathrm{L}}_{\mathrm{Q}\mathrm{G}}$	$\:{\mathrm{L}}_{\mathrm{t}}$	$\:{\mathrm{L}}_{\mathrm{t}\mathrm{s}}$	$\:{\mathrm{S}}_{\mathrm{g}1}$	$\:{\mathrm{S}}_{\mathrm{g}2}$
Value (mm)	2.26	0.66	16	8	0.92	3.92	2.08	2.04	1.8

## Parametric study for optimal design parameters

The parametric study of the proposed antenna is essential for understanding the electromagnetic behaviour governing the dual-band circularly polarized operation at 28 GHz and 38 GHz. Since the antenna geometry is based on a modified quatrefoil radiating structure combined with capacitive-loaded parasitic elements and defected ground features, the resonance characteristics and polarization purity are strongly dependent on several critical geometrical parameters. Therefore, a systematic investigation of these parameters is necessary to identify their individual contributions to resonance tuning, impedance matching, bandwidth enhancement, and axial ratio performance.

**Fig. 2:**
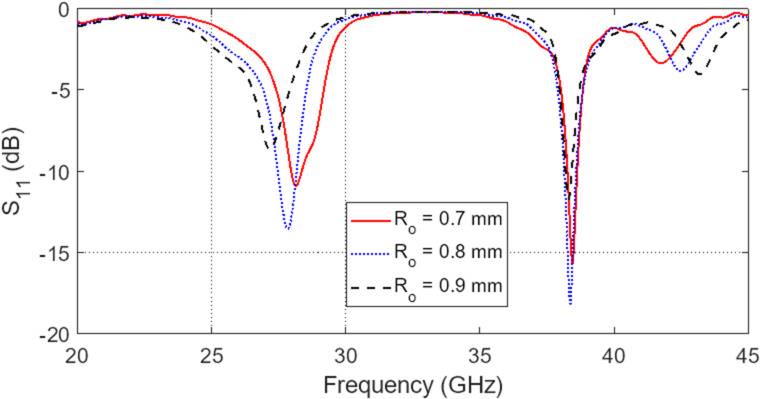
Simulated reflection coefficient $\:{S}_{11}$ for different values of the quatrefoil lobe radius $\:{R}_{o}$, showing its effect on the lower-band resonance around 28 GHz.

For the lower operating band centered around 28 GHz, the resonance frequency is primarily controlled by the dimensions of the main radiating patch. In particular, the lobe radius $\:{R}_{o}\:$of the quatrefoil geometry plays a dominant role in determining the effective current path length of the radiating structure. Any variation in $\:{R}_{o}$ directly modifies the electrical length of the patch, leading to a noticeable shift in the lower-band resonance location. Increasing the lobe radius enlarges the current circulation path and consequently shifts the resonance toward lower frequencies, whereas reducing $\:{R}_{o}$ moves the resonance upward. In addition to the lobe radius, the separation distance between the two axial lobes or circles of the quatrefoil structure, denoted as $\:{S}_{Q}$, significantly influences the patch size. This parameter modifies the effective resonant cavity dimensions at the 28 GHz resonance. Both parameters $\:{R}_{o}$ and $\:{S}_{Q}$ are the main parameters that determines the lower-band impedance response and strongly affects the matching characteristics around the 28 GHz band. The influence of the lobe radius $\:{R}_{o}$ on the antenna response is illustrated in Fig. 2, where the resonance shift associated with variations in the effective radiating length can be clearly observed.

On the other hand, the resonance behavior associated with the upper operating band near 38 GHz is mainly governed by the parasitic elements integrated around the primary radiator. These parasitic structures are capacitively loaded and strategically positioned to introduce an additional resonant mode at higher frequencies. The size, shape, and placement of these parasitic elements determine the coupling strength between the main radiator and the surrounding resonators, which directly controls the location and bandwidth of the 38 GHz resonance. Variations in the dimensions of the parasitic elements modify their effective capacitance coupling, resulting in a shift of the higher-frequency resonance. Furthermore, the spatial positioning of these elements relative to the main patch affects the near-field interaction and surface current distribution, making their location another critical parameter for accurate tuning of the upper band.

**Fig. 3:**
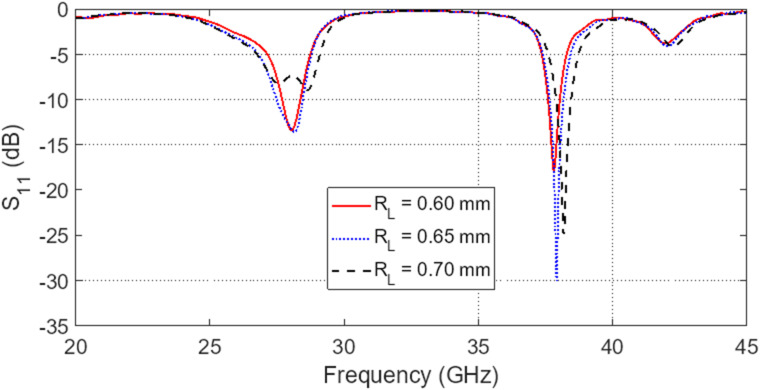
Simulated reflection coefficient $\:{S}_{11}$ for different values of the parameter $\:{R}_{L}$, representing the radius of the two half-circle ring parasitic elements, illustrating its effect on the upper-band resonance around 38 GHz.

**Fig. 4:**
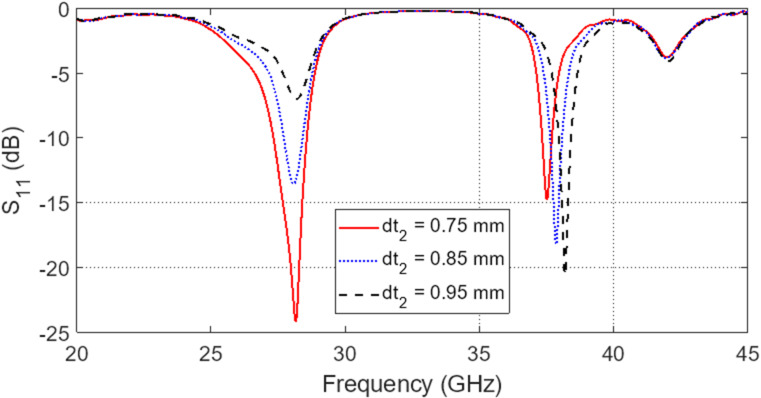
Simulated reflection coefficient $\:{S}_{11}$ for different values of the parameter $\:{d}_{t2}$, representing the side length of the triangular parasitic elements, showing its influence on the upper-band resonance characteristics around 38 GHz.

The parametric influence of the capacitive-loaded parasitic elements is investigated through two major geometrical variables. Figure 3 presents the effect of the parameter $\:{R}_{L}$, which represents the radius of the two half-circle ring parasitic elements. Changing $\:{R}_{L}$ alters the equivalent resonant path and capacitive coupling strength of the parasitic resonators, thereby controlling the resonance characteristics of the 38 GHz band. In addition, Fig. 4 studies the parameter $\:{d}_{t2}$, defined as the side length of the triangular parasitic elements. Variations in $\:{d}_{t2}$, modify the electromagnetic interaction between the triangular parasitic structures and the main radiator, producing noticeable effects on the upper-band impedance response and resonance stability. Through careful optimization of $\:{R}_{L}$ and $\:{d}_{t2}$, stable resonance behavior and enhanced impedance bandwidth at 38 GHz can be achieved.

In addition to impedance characteristics, the circular polarization performance of the antenna is highly dependent on the geometrical axial symmetry of the overall structure. The axial ratio at both operating bands is mainly controlled by the axial symmetry of the antenna configuration, which ensures the generation of two orthogonal modes with nearly equal amplitudes and a quadrature phase difference. The symmetric arrangement of the quatrefoil patch, together with the balanced distribution of the parasitic elements, contributes significantly to maintaining stable circular polarization across both frequency bands. Moreover, the defected ground structure containing axially symmetric perforations plays a crucial role in refining the polarization characteristics. These symmetric ground perforations modify the surface current distribution beneath the radiator and help balance the orthogonal field components required for circular polarization operation. Any asymmetry introduced into the radiator or ground plane can disturb the phase and amplitude balance between orthogonal modes, thereby degrading the axial ratio performance. Consequently, maintaining precise axial symmetry in both the radiator and the ground perforation design is essential for achieving low axial ratio values and stable circular polarization at both 28 GHz and 38 GHz.

Finally, the optimized antenna design is achieved through extensive parametric simulations in which all antenna dimensions and structural parameters are jointly optimized to obtain the desired dual-band circularly polarized operation. The optimization accounts for the interaction between the quatrefoil radiator, capacitive-loaded parasitic elements, and axially symmetric ground perforations, since each parameter affects both the resonance behavior and axial ratio performance. Special attention is given to accurately tuning the resonances at 28 GHz and 38 GHz while maintaining good impedance matching and low axial ratio values at both bands. Only representative examples of the most influential parameters are presented for clarity. However, the complete antenna design and final dimensions were obtained through extensive full-wave parametric simulations and iterative optimization of all antenna parameters.

## Two-port MIMO antenna design with polarization diversity

**Fig. 5:**
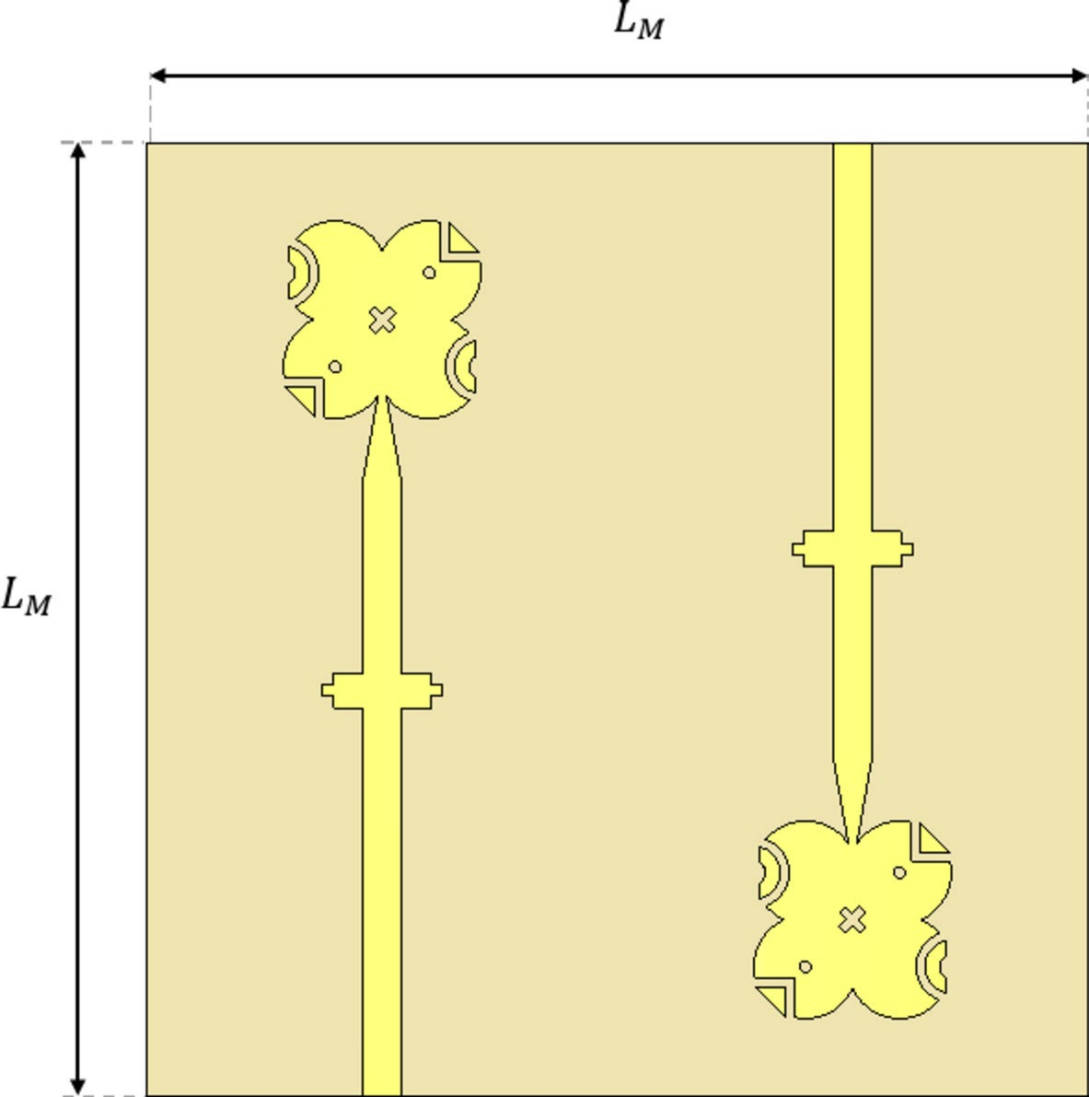
Geometry of the proposed two-port MIMO antenna system with dual circularly polarized 28/38 GHz.

The antenna proposed in Sect.  2 is used to design a two port multiple-input multiple-output (MIMO) antenna for 5G and beyond applications. The proposed millimeter-wave MIMO system consists of two closely integrated circularly polarized antenna elements with opposite polarization senses, where one antenna operates with left-hand circular polarization (LHCP) and the other with right-hand circular polarization (RHCP). The MIMO configuration is implemented using the antenna element designed and analyzed in the previous sections, where two identical antenna structures are arranged to form a two-port MIMO system with opposite circular polarization senses. The complete MIMO configuration occupies a compact footprint of 16 × 16 mm², making it suitable for integration into space-limited platforms such as 5G user equipment and compact wireless modules. The proposed MIMO system is illustrated in Fig. 5, where the overall dimension parameter $\:{L}_{M}$ is indicated as 16 mm, representing the total side length of the square MIMO layout. When port 1 is excited, the antenna generates RHCP radiation, whereas excitation of port 2 results in LHCP radiation. Both antenna elements are designed to operate at the 28 GHz and 38 GHz millimeter-wave bands, which are widely considered key frequency allocations for high-capacity fifth-generation and beyond wireless communication systems. The use of orthogonal circular polarizations within the same MIMO structure provides significant performance advantages compared with conventional linearly polarized MIMO arrays. Specifically, the LHCP and RHCP elements offer strong polarization diversity, which naturally reduces mutual coupling and correlation between the antenna ports even when the elements are placed in close proximity within a compact layout. This polarization orthogonality contributes to improved isolation and a low envelope correlation coefficient (ECC), which are essential parameters for efficient MIMO operation. Furthermore, circular polarization enhances the robustness of the wireless link against polarization mismatch and multipath propagation effects that commonly occur due to arbitrary device orientation in practical scenarios. The dual-band operation at 28/38 GHz further increases the flexibility of the system by enabling multi-band communication capability while maintaining a small physical size. Consequently, the proposed dual circularly polarized MIMO configuration provides enhanced channel capacity, improved link reliability, and effective spatial and polarization diversity without increasing the antenna footprint, making it a promising candidate for high-data-rate millimeter-wave applications in future 5G and beyond wireless communication systems.

## Modified quatrefoil MIMO fabrication and measured performance

**Fig. 6:**
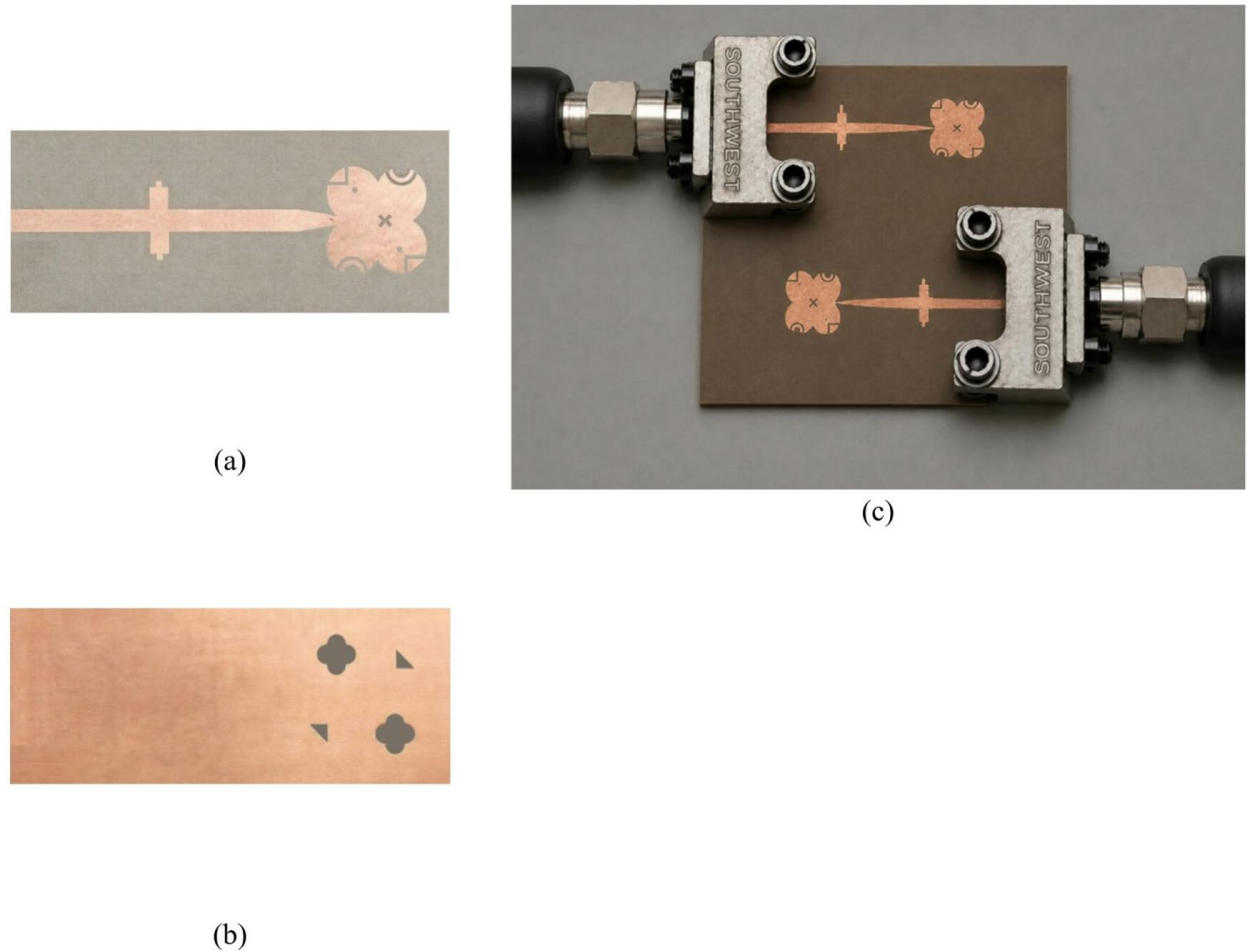
(**a**) Front and (**b**) Back views of the fabricated modified quatrefoil-shaped antenna, (**c**) Two-port MIMO antenna system.

**Fig. 7:**
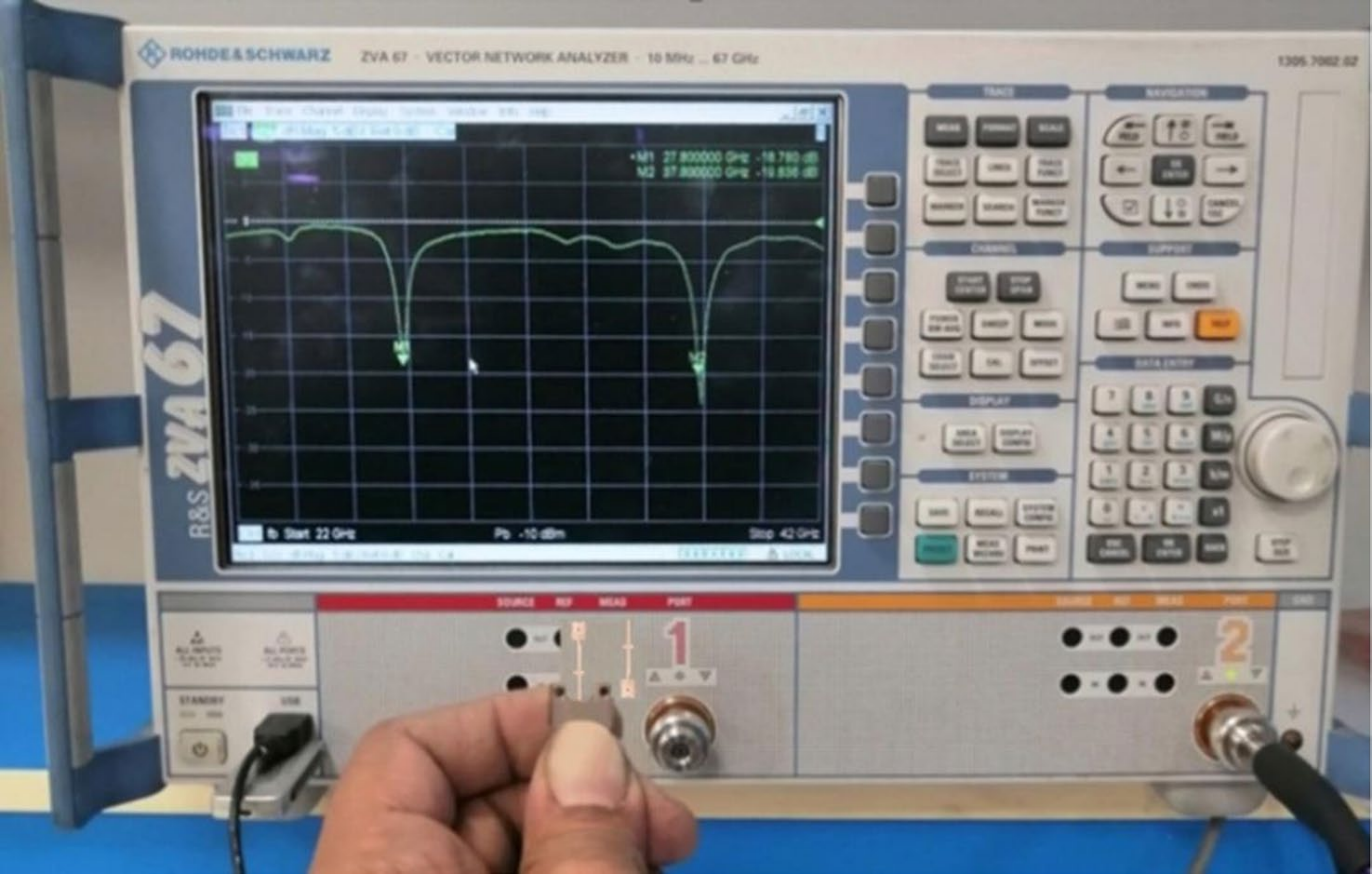
Experimental setup of the fabricated MIMO antenna during reflection coefficient measurements using the Rohde & Schwarz ZVA67 vector network analyzer (VNA).

**Fig. 8:**
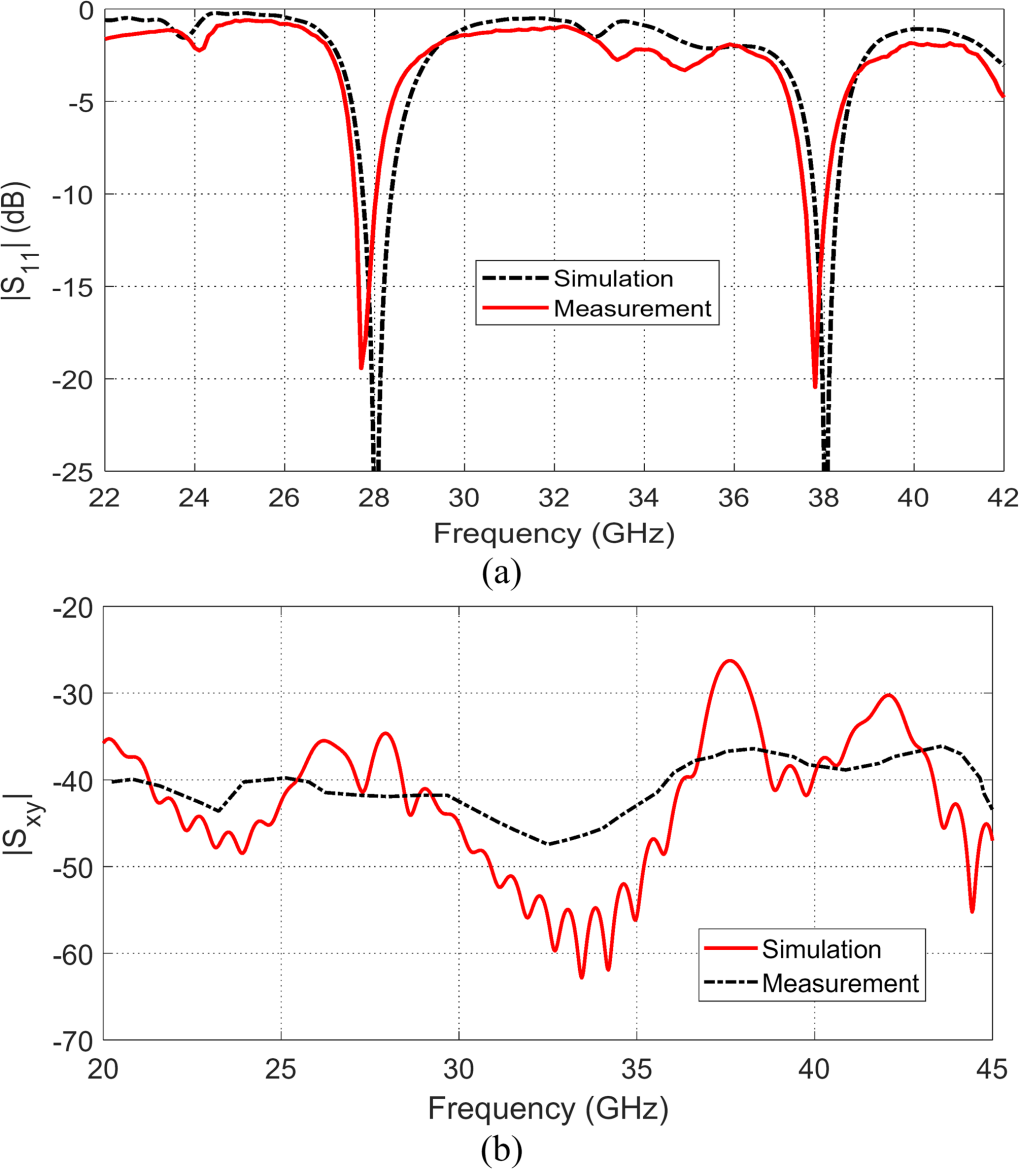
**a**: Simulated and measured reflection coefficient (S_xx_) of the proposed two-port MIMO antenna. **b**: Simulated and measured coupling coefficient (S_xy_) of the proposed two-port MIMO antenna.

The proposed two-port MIMO antenna was successfully fabricated on the Rogers RO3003™ substrate and experimentally characterized to validate the simulated performance. Figure 6a and b show the top and bottom views of the fabricated single antenna element, illustrating the realized radiating structure and ground-plane configuration. The complete fabricated MIMO prototype is presented in Fig. 6c, where the arrangement of the two antenna elements and the overall compact structure can be clearly observed. To verify the antenna performance, measurements were carried out using a Rohde & Schwarz ZVA67 vector network analyzer (VNA). The experimental measurement setup is depicted in Fig. 7, showing the fabricated MIMO antenna connected to the VNA during characterization. The measured reflection coefficient (S_11_) results are presented in Fig. 8a, and the mutual coupling coefficient is shown in Fig. 8b. The measured response demonstrates good impedance matching over the desired operating bands and shows close agreement with the simulated results, confirming the validity of the proposed MIMO antenna design. Minor discrepancies between the simulated and measured responses may be attributed to fabrication tolerances, connector losses, and measurement uncertainties at millimeter-wave frequencies.

**Fig. 9:**
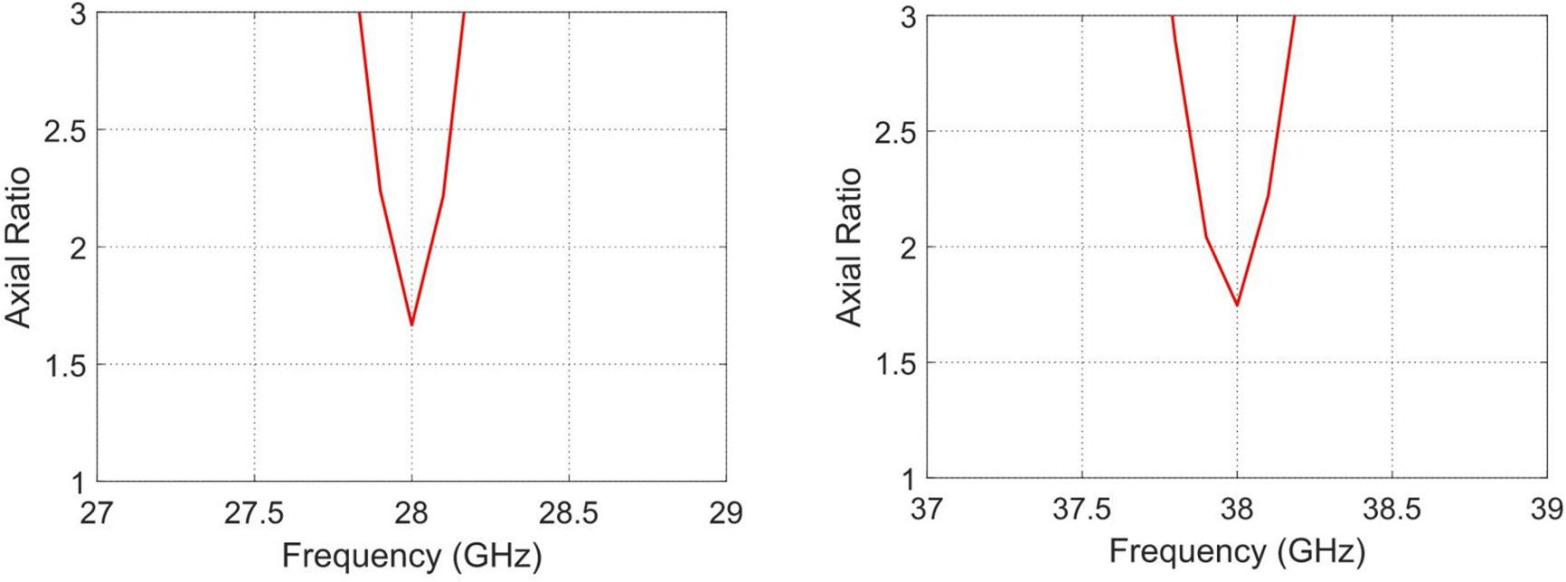
Axial ratio of the proposed modified quatrefoil MIMO antenna, demonstrating circular polarization with values below 3 dB at 28 GHz and 38 GHz.

In addition, the circular polarization performance of the proposed MIMO antenna is evaluated through the axial ratio characteristics shown in Fig. 9. The axial ratio results exhibit values below 3 dB within the operating bands, confirming the achievement of effective circular polarization performance with good agreement between the simulated and measured results.

## Radiation pattern characteristics of the two-port MIMO antenna

**Fig. 10:**
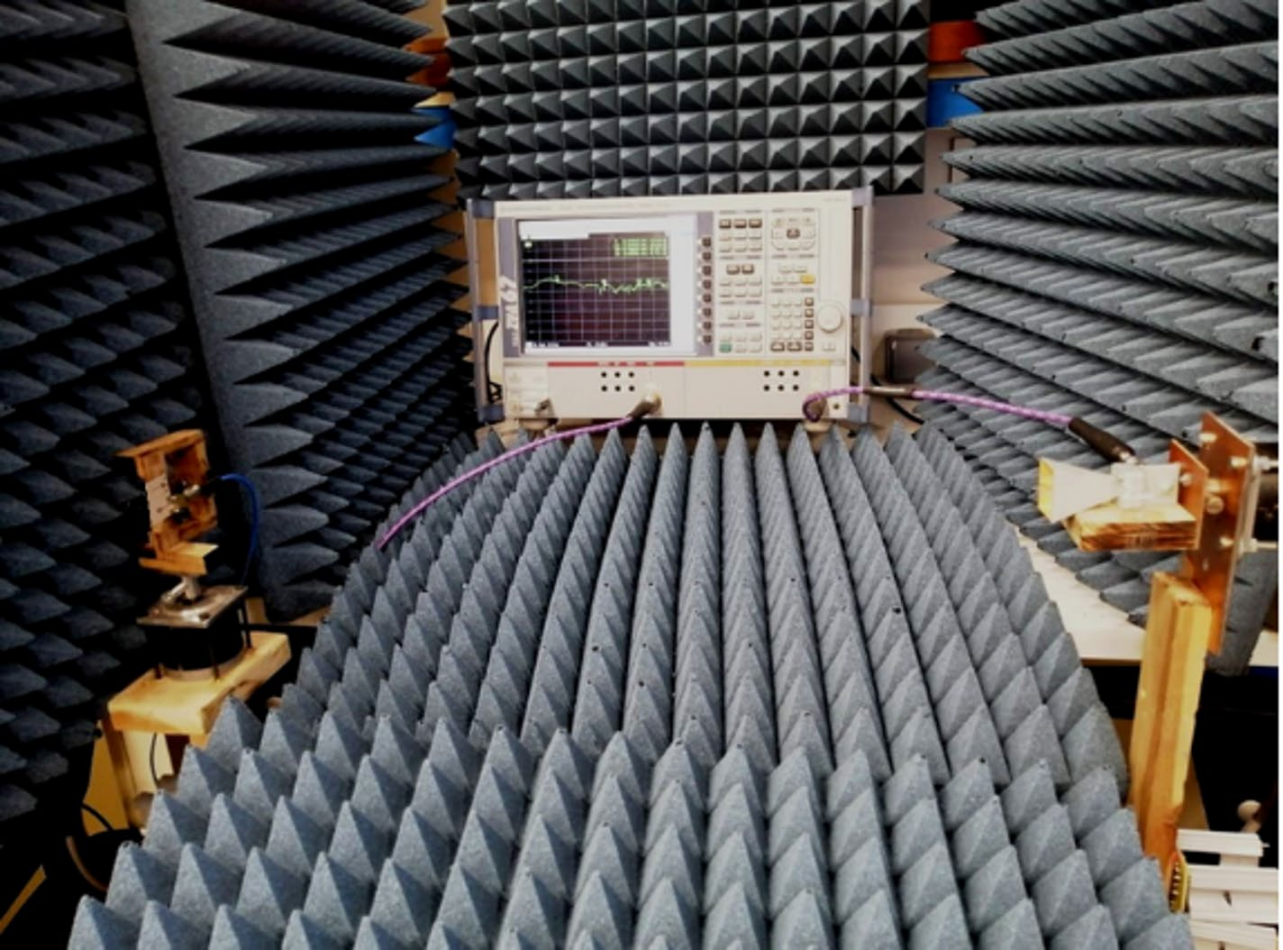
Radiation and gain pattern measurement setup showing the AUT and reference horn antenna connected to ports 1 and 2 of the VNA, respectively.

The radiation pattern and gain measurements of the proposed antenna were carried out using the experimental setup shown in Fig. 10. The measurements were performed inside an anechoic chamber to minimize reflections and external electromagnetic interference. A standard reference horn antenna model A-INFO LB-180,400–2.4 F was positioned in a boresight configuration facing the antenna under test (AUT). The AUT was mounted on a motorized rotator that enables accurate angular rotation in both the azimuth and elevation planes for complete far-field characterization. To ensure reliable measurements, the distance between the AUT and the reference horn antenna satisfied the far-field condition.

The gain pattern measurements for both the single-element antenna and the proposed MIMO antenna were conducted using a vector network analyzer (VNA), where the AUT and the reference horn antenna were connected to ports 1 and 2 of the VNA, respectively. During the measurement process, the AUT was rotated in discrete angular steps of 10° over a full 360° scan, while the transmission coefficient magnitude |S_21_| was automatically recorded at each angular position. The measured data were then processed and normalized to obtain the radiation patterns in the principal planes.

**Fig. 11:**
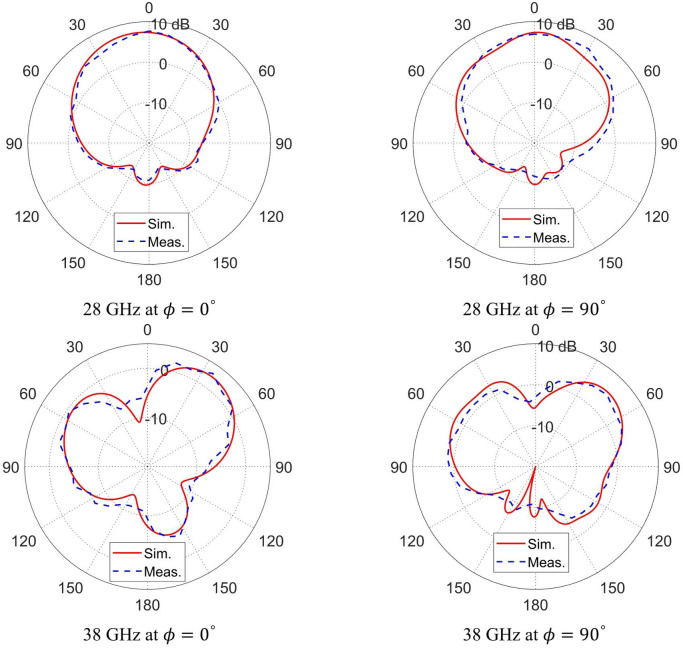
Simulated and measured radiation patterns of the two-port MIMO when port 1 is excited, at both frequencies 28 and 38 GHz in the $\:\phi\:={0}^{^\circ\:}$ and $\:\phi\:={90}^{^\circ\:}$ planes.

The far-field radiation patterns measured at 28 GHz and 38 GHz in the principal planes (ϕ = 0° and ϕ = 90°) are illustrated in Fig. 11. The obtained results demonstrate stable broadside radiation characteristics with good agreement between the simulated and measured patterns. Moreover, the radiation patterns exhibit symmetrical main lobes with minimal distortion, confirming stable directional radiation and reliable circular polarization performance across both operating bands. These results validate the suitability of the proposed antenna for dual-band millimeter-wave MIMO communication applications requiring stable radiation characteristics and polarization performance.

## Surface current distribution for CP behavior

The surface current distribution is analyzed to further explain and validate the circular polarization performance of the proposed two-port MIMO antenna. In this analysis, Port 1 of the MIMO structure is excited, and the surface current distributions are investigated at the operating frequencies of 28 GHz and 38 GHz. To provide a clear understanding of the circular polarization mechanism, the current distributions are observed at four sequential phase instants of the excitation cycle: 0°, 90°, 180°, and 270°. At these different phases, the surface current vectors rotate gradually around the modified quatrefoil-shaped radiator, completing a full circular rotation. The continuous temporal rotation of the current vectors indicates the existence of a rotating electric field in the far-field region, which confirms the generation of circular polarization. This rotational current behavior is evident at both 28 GHz and 38 GHz, demonstrating that circular polarization is successfully achieved in both operating bands of the proposed MIMO antenna.

Nevertheless, noticeable differences can be observed between the current distributions at the two resonant frequencies. At 28 GHz, the surface current distribution corresponds to a first-order resonant mode, where a dominant current loop rotates smoothly and uniformly along the radiator surface. On the other hand, the current distribution at 38 GHz still preserves the rotational characteristic required for circular polarization, but the current profile represents a second-order resonant mode. In this case, additional current variations and localized current nulls appear across the radiator structure, resulting in a more complex current configuration. The presence of these higher-order current components at 38 GHz explains the different resonant behavior between the two frequency bands while maintaining circular polarization performance. These results demonstrate the capability of the modified quatrefoil-shaped geometry to support circularly polarized radiation through multiple resonant modes within the proposed MIMO configuration.

**Fig. 12:**
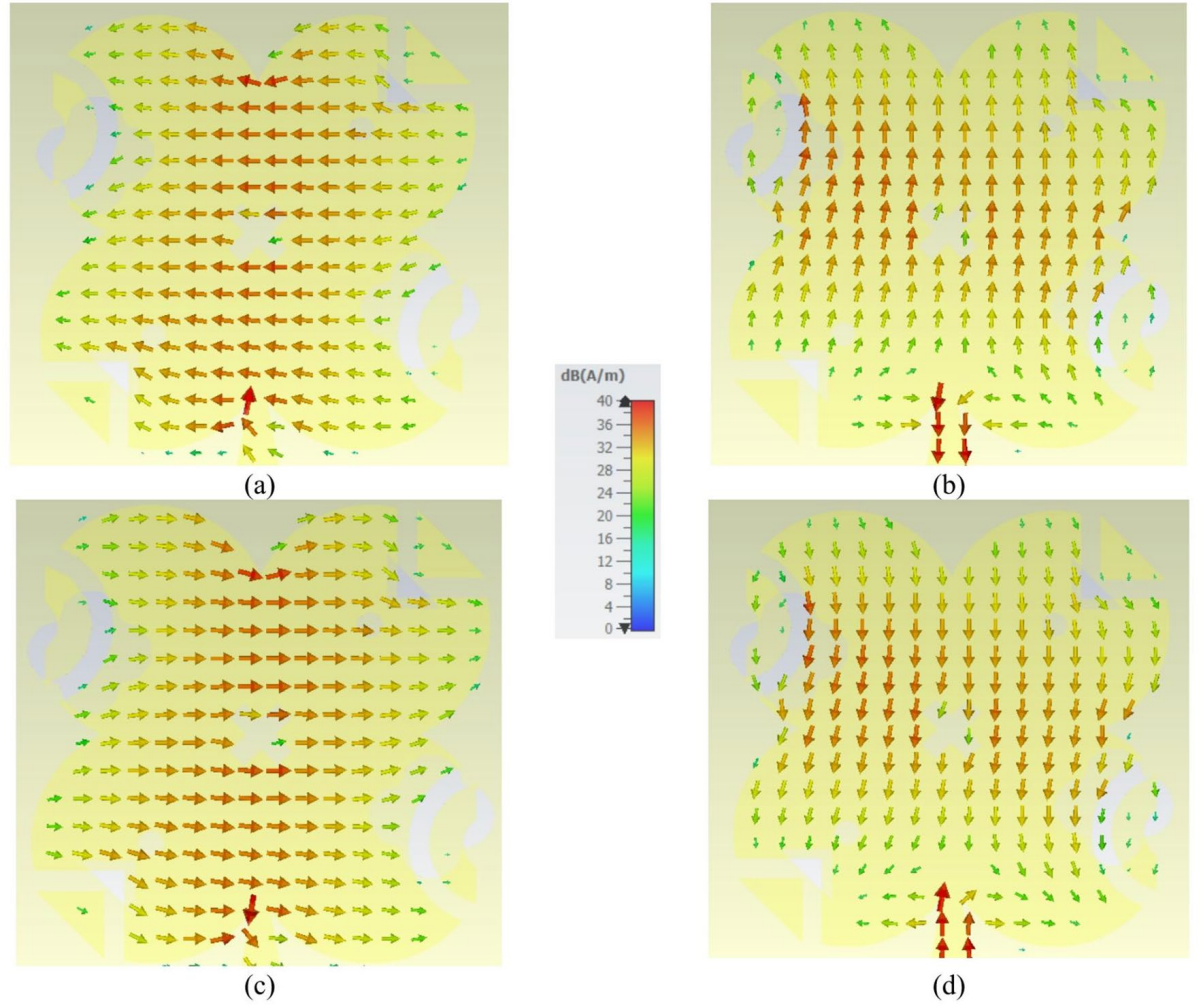
Surface current distributions of the proposed two-port MIMO antenna at 28 GHz for Port 1 excitation at phase instants of (**a**) 0°, (**b**) 90°, (**c**) 180°, and (**d**) 270°, over one circular polarization cycle.

**Fig. 13:**
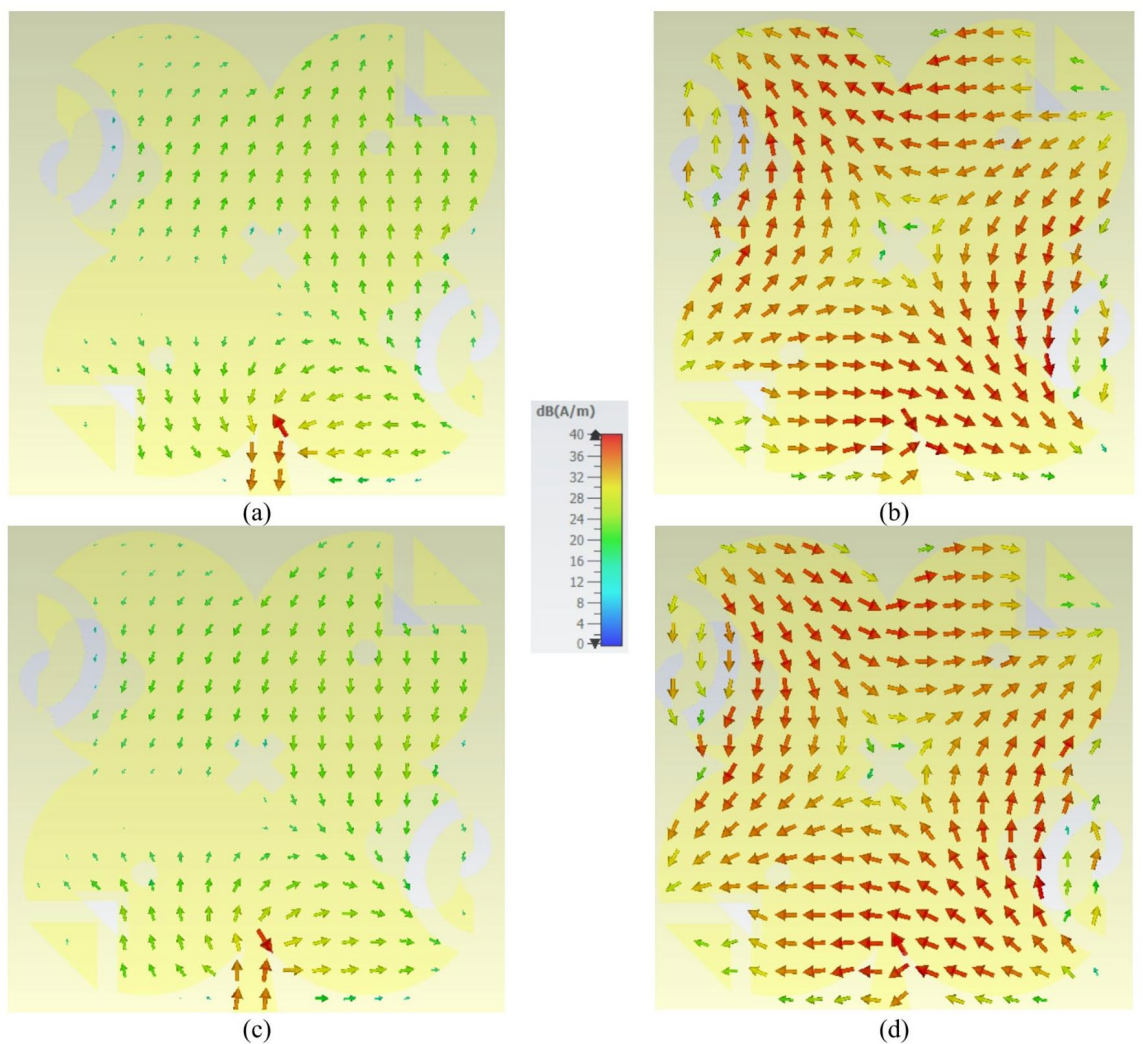
Surface current distributions of the proposed two-port MIMO antenna at 38 GHz for Port 1 excitation at phase instants of (**a**) 0°, (**b**) 90°, (**c**) 180°, and (**d**) 270°, over one circular polarization cycle.

As shown in Fig. 12, the surface current distributions at 28 GHz illustrate a first-order resonant mode, where four separate current snapshots correspond to the quarter-cycle phase instants of 0°, 90°, 180°, and 270°. The current rotation is smooth and consistent around the radiator, confirming dominant fundamental-mode operation. Similarly, Fig. 13 presents the surface current distributions at 38 GHz for the same four phase instants. Although the rotating current behavior at 38 GHz still verifies circular polarization operation, the current paths become more complicated due to the excitation of a second-order resonant mode with additional current variations across the radiator surface. Overall, these observations confirm that the proposed modified quatrefoil-shaped two-port MIMO antenna is capable of achieving circular polarization at both 28 GHz and 38 GHz while operating with different resonant current modes at the two frequency bands.

## MIMO performance metrics

**Fig. 14:**
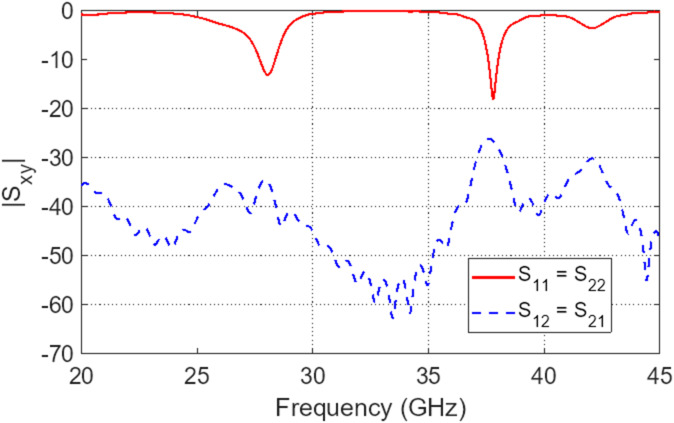
S-parameters of the proposed two-port MIMO antenna.

The two-port MIMO antenna demonstrates excellent impedance matching and isolation characteristics across the targeted millimeter-wave bands. The self-parameters (S₁₁ and S₂₂) of the antenna show good matching at both 28 GHz and 38 GHz, indicating that each antenna element efficiently radiates the input power with minimal reflection. Simultaneously, the mutual coupling parameters (S₁₂ and S₂₁) remain below − 25 dB across the operating bands, confirming a high degree of isolation between the two circularly polarized ports despite the compact 16 × 16 mm² layout. This low mutual coupling is a direct result of the orthogonal LHCP and RHCP configuration, which reduces interference and correlation between the ports, thereby improving MIMO performance. These key S-parameter characteristics of the proposed system are illustrated in Fig. 14, clearly showing both the dual-band matching and the excellent port isolation essential for reliable dual-port MIMO operation.

**Fig. 15:**
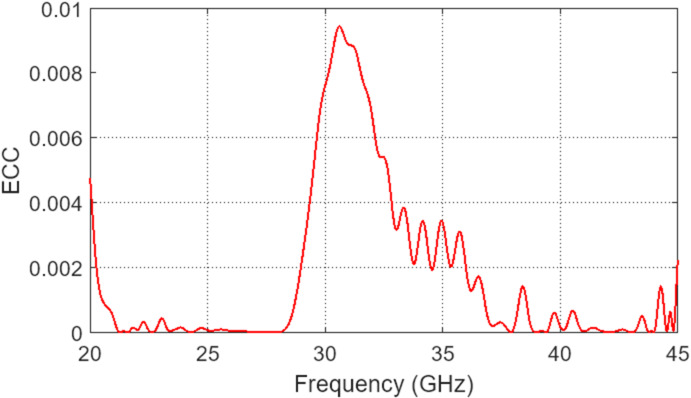
Envelope correlation coefficient (ECC) of the proposed two-port MIMO antenna, showing values near zero at 28 GHz and 38 GHz, indicating excellent diversity performance.

The envelope correlation coefficient (ECC) is a key metric in MIMO systems, defined as a measure of the correlation between the radiation patterns of different antenna ports. It indicates how similar the signals received or transmitted by multiple antennas are, with an ECC of 0 representing completely uncorrelated signals and an ECC close to 1 indicating highly correlated, less effective diversity performance. Low ECC values are critical for achieving high diversity gain and maximizing MIMO channel capacity. In the proposed two-port MIMO antenna, the ECC was calculated using CST Microwave Studio based on the simulated far-field radiation patterns. The results show that the ECC remains near zero across both the 28 GHz and 38 GHz operating bands, confirming excellent diversity performance and effective isolation between the LHCP and RHCP ports. These results are illustrated in Fig. 15, highlighting the capability of the proposed MIMO configuration to provide robust dual-band operation with minimal signal correlation.

**Fig. 16:**
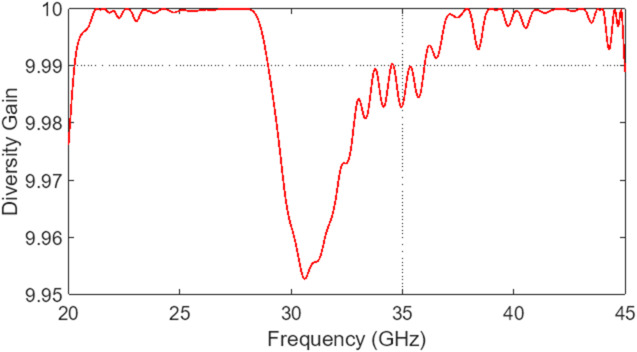
Diversity gain (DG) of the proposed two-port MIMO antenna, showing values near 10 dB at 28 GHz and 38 GHz, indicating excellent diversity performance.

Diversity gain (DG) is an important performance metric in MIMO antenna systems, as it quantifies the improvement in signal reliability and quality achieved by combining signals from multiple antenna ports under multipath propagation conditions. DG reflects the ability of the MIMO system to mitigate fading effects, which are common at millimeter-wave frequencies due to reflections and scattering. A higher DG indicates better performance in maintaining a stable communication link, with an ideal value close to 10 dB for a two-port MIMO configuration, representing near-optimal diversity performance. In the proposed two-port MIMO antenna, the DG was calculated using simulated far-field radiation patterns, and the results demonstrate that the DG remains almost 10 dB across both the 28 GHz and 38 GHz operating bands. This confirms that the antenna system effectively exploits spatial and polarization diversity to enhance signal robustness and reliability. Figure 16 shows the diversity gain (DG) of the proposed MIMO antenna, highlighting its excellent performance across the dual-band operation.

**Fig. 17:**
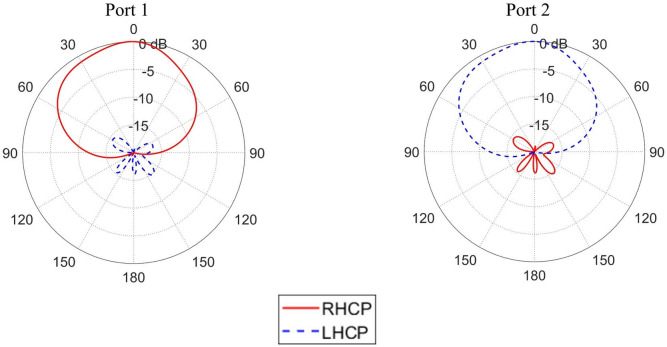
Left-hand and right-hand radiation patterns at $\:\phi\:={0}^{^\circ\:}$ plane for 28 GHz for Port 1 and Port 2.

**Fig. 18:**
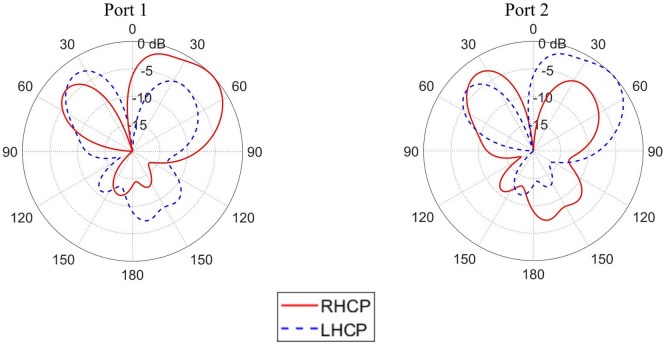
Left-hand and right-hand radiation patterns at $\:\phi\:={0}^{^\circ\:}$ plane for 38 GHz for Port 1 and Port 2.

**Fig. 19:**
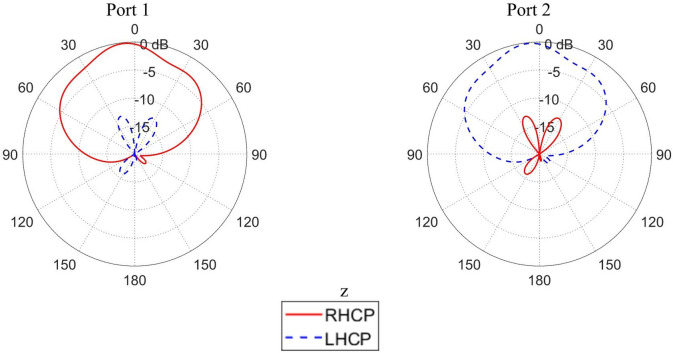
Left-hand and right-hand radiation patterns at $\:\phi\:={90}^{^\circ\:}$ plane for 28 GHz for Port 1 and Port 2.

**Fig. 20:**
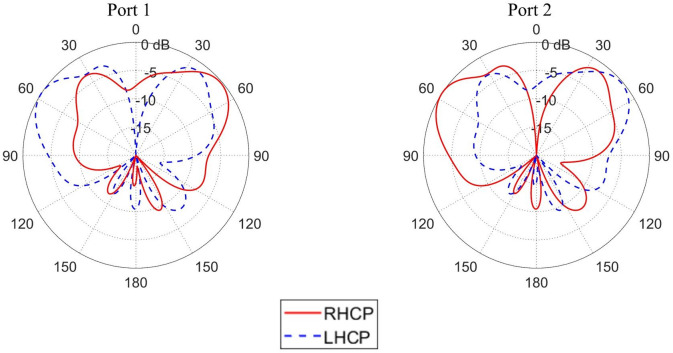
Left-hand and right-hand radiation patterns at $\:\phi\:={90}^{^\circ\:}$ plane for 38 GHz for Port 1 and Port 2.

The left-hand and right-hand radiation patterns of the proposed MIMO antenna system were calculated to evaluate the circular polarization characteristics of both antenna elements at the two operating frequencies. The radiation patterns were analyzed in the principal planes at $\:\phi\:={0}^{^\circ\:}$ and $\:\phi\:=9{0}^{^\circ\:}$, and the results are presented in Figs. 17, 18, 19 and 20 for both ports of the MIMO structure. Specifically, Fig. 17 illustrates the radiation patterns at $\:\phi\:={0}^{^\circ\:}$ for 28 GHz for Port 1 and Port 2, while Fig. 18 presents the corresponding patterns at $\:\phi\:={0}^{^\circ\:}$ for 38 GHz for both ports. In addition, Fig. 19 shows the radiation patterns at $\:\phi\:={90}^{^\circ\:}$ for 28 GHz for Port 1 and Port 2, whereas Fig. 20 depicts the radiation patterns at $\:\phi\:={90}^{^\circ\:}$ for 38 GHz for both ports. In each case, the left-hand circularly polarized (LHCP) and right-hand circularly polarized (RHCP) components are plotted to verify the polarization behavior of the antenna elements. The obtained radiation patterns demonstrate that the proposed antenna maintains good circular polarization performance at both operating frequencies and in both principal planes, confirming the effectiveness of the design for dual-band millimeter-wave MIMO applications.

## Comparison with published work

To highlight the effectiveness of the proposed antenna, its performance is systematically compared with several recent dual-band 28/38 GHz antenna designs reported in the literature, focusing on key metrics such as antenna size, polarization characteristics, radiation efficiency, and realized gain at each operating band, as summarized in Table 2.

**Table 2:** Comparison of the proposed dual-band 28/38 GHz circularly polarized antenna with recent antenna designs reported in the literature.

[9]	Antenna Size ($\:\mathrm{m}\mathrm{m}\times\:\mathrm{m}\mathrm{m}$)	Polarization	Gain at 28/38 (dBi)	Efficiency at 28/38 (%)	Operating bandwidth (GHz)	Isolation (dB)	axial ratio bandwidth at 28/38 (GHz)	ECC at 28/38 GHz
	$\:2\times\:10$	Circular	6.8/5.9	85/80	27.6–28.3 35.2–47.2	NA	NA	NA
[10]	$\:20.4\times\:26.4$	Circular	6.2/5.9	92/90	27.2–29.2 36–40	36/36	27.82–29.08 37.3–38.23	$\:3\times\:{10}^{-7}/5\times\:{10}^{-7}$
[11]	$\:26\times\:26$	Linear	5.56 dBi (w/o AMC) 8.23 dBi (with AMC	NA	27.3–30.9 34.9–40	26/27	NA	0.05/0.04
[12]	$\:9.69\times\:8.25$	Linear	8.3/4.9	91/84	27.5–29.5 32.6–44.3	20/20	NA	NA
[13]	$\:26\times\:26$	Linear	7.4/5.2	88.8/88	27.7–28.3 36.2–39.1	24/26	NA	NA
Present Work	$\:16\times\:16$	Circular	7.5/5.5	87/82	27.6–28.4 37.5–38.3	35/27	27.6–28.18 37.75–38.25	$\:8\times\:{10}^{-6}/1.3\times\:{10}^{-5}$

The antenna designs listed in Table 2 demonstrate different design philosophies and performance trade-offs for dual-band 28/38 GHz operation. Ref^13^. achieves circular polarization at both frequency bands using geometrical perturbations, resulting in a compact structure; however, the reported gain and efficiency at 38 GHz are relatively moderate. Ref^14^. achieves circular polarization at 28 GHz and 38 GHz, but with large size microstrip patch. In Ref^15^., an AMC/metasurface-backed configuration is employed to enhance gain and axial-ratio bandwidth, particularly at 38 GHz, at the expense of increased antenna size, structural complexity, and fabrication cost. Ref^16^. presents a compact dual-band antenna with high gain and efficiency, yet it operates with linear polarization, making it more sensitive to polarization mismatch in practical millimeter-wave scenarios. Ref^17^. offers a mature dual-band solution with good efficiency and relatively high gain; however, it also relies on linear polarization and occupies a larger footprint. The proposed antenna achieves circular polarization at both 28 GHz and 38 GHz while maintaining a very compact size (8 × 16 mm²) and avoiding the use of bulky reflectors or metasurfaces. Moreover, it provides clearly reported and competitive performance metrics, including realized gains of 7.5 dBi at 28 GHz and 5.5 dBi at 38 GHz, along with high radiation efficiencies of 87% and 82%, respectively. These features collectively highlight the proposed design as a well-balanced and practically viable solution for compact 5G and future millimeter-wave user equipment.

## Conclusion

The paper presented a compact dual-band circularly polarized two-port MIMO antenna designed for millimeter-wave applications at 28 and 38 GHz. The proposed single-element antenna occupies an ultra-small area of 8 mm × 16 mm and is fabricated on a Rogers RO3003 substrate with a dielectric constant of 3 and a thickness of 0.25 mm. The antenna successfully achieves circular polarization at both operating frequencies, with measured axial ratios of 1.4 dB at 28 GHz and 2.2 dB at 38 GHz, which helps mitigate polarization mismatch in practical wireless environments. In addition, the antenna provides peak realized gains of 7 dBi at 28 GHz and 4.5 dBi at 38 GHz. The measured impedance matching and radiation patterns show good agreement with the simulated results, confirming the validity of the proposed design. Based on the verified single-element antenna, a two-port MIMO configuration was implemented, where the two elements produce opposite circular polarizations (RHCP and LHCP), enabling polarization diversity and improving the overall system performance. The MIMO configuration demonstrates low envelope correlation coefficient and high diversity gain, indicating effective isolation and diversity characteristics. Due to its compact size, dual-band circular polarization capability, and favorable MIMO diversity performance, the proposed antenna is a promising candidate for space-constrained 5G and future millimeter-wave wireless communication devices.

## Data Availability

The datasets used and/or analyzed during the current study available from the corresponding author on reasonable request.
